# Supplementary material for: Compound marine heatwaves and ocean acidity extremes
Source: Nat Commun. 2022 Aug 16;13:4722. doi: 10.1038/s41467-022-32120-7 (PMC9381716; doi:10.1038/s41467-022-32120-7)
Supplement: Supplementary file 1 — Supplementary Information [file 41467_2022_32120_MOESM1_ESM.pdf]

Supplementary information for

Compound marine heatwaves and ocean acidity extremes

Friedrich A. Burger[1,2*], Jens Terhaar[1,2], Thomas L. Frölicher[1,2]

[1]*Climate and Environmental Physics, Physics Institute, University of Bern, Bern, Switzerland.*

[2]*Oeschger Centre for Climate Change Research, University of Bern, Bern, Switzerland.*

* *corresponding author: friedrich.burger@unibe.ch*

	LMF				p-value[2]	
	Time series (monthly[1])	Time series (daily mean)	Gridded data (monthly mean)	GFDL ESM2M (daily mean)	Gridded data	GFDL ESM2M
**KNOT-K2**	1.2 (0.1 – 4.4)		1.2 (0.5 – 2.2)	1.4 (1.4 – 1.4)	1	1
**HOT**	1.0 (0.3 – 2.4)		4.6 (3.4 – 5.8)	4.4 (4.3 – 4.4)	0.0005*	0.00003*
**BATS**	3.7 (2.5-5.0)		4.2 (3.0 – 5.4)	3.9 (3.9 – 3.9)	0.53	0.65
**ESTOC**	5.0 (3.2 – 6.8)		2.8 (1.8 – 4.0)	3.5 (3.5 – 3.6)	0.07	0.14
**137 °E 30 °N**	2.9 (0.8 –5.8)		3.4 (2.3 – 4.6)	3.5 (3.5 – 3.6)	1	0.75
**137 °E 5 °N**	2.6 (0.5 – 6.0)		1.6 (0.8 – 2.7)	2.5 (2.4 – 2.5)	0.60	1
**Munida**	3.0 (1.1 – 5.6)		1.0 (0.4 – 2.0)	1.3 (1.3 – 1.4)	0.09	0.10
**CARIACO**	4.2 (2.3 – 6.3)		2.0 (1.1 – 3.1)	2.3 (2.3 – 2.3)	0.12	0.10
**KEO**	6.1 (3.1 – 8.2)	4.5 (3.9 – 5.0)	3.0 (1.9 – 4.2)	3.4 (3.3 – 3.4)	0.06	0.08
**Papa**	1.0 (0.1 – 3.9)	0.9 (0.6 – 1.3)	0.0 (0.0 – 0.6)	2.4 (2.4 – 2.5)	0.16	0.46
**STRATUS**	3.1 (0.9 – 6.1)	3.5 (3.0 – 4.0)	1.2 (0.5 – 2.2)	4.1 (4.1 – 4.2)	0.14	0.74
**TAO125W**	0.0 (0.0 – 2.4)	0.0 (0.0 – 0.2)	0.0 (0.0 – 0.6)	0.1 (0.1 – 0.1)	1	1
**TAO140W**	0.0 (0.0 – 2.8)	0.1 (0.0 – 0.3)	0.0 (0.0 – 0.6)	0.1 (0.1 – 0.1)	1	1
**TAO170W**	0.0 (0.0 – 3.4)	0.0 (0.0 – 0.2)	0.0 (0.0 – 0.6)	0.7 (0.7 – 0.7)	1	1
**WHOTS**	3.4 (1.3 – 6.1)	3.2 (2.7 – 3.7)	4.6 (3.4 – 5.8)	4.4 (4.3 – 4.4)	0.53	0.56

[1]For stations KNOT-K2, HOT, BATS, ESTOC, 137 °E 30 °N, 137°E 5 °N, Munida, and CARIACO, point measurements on roughly monthly resolution were used, for the remaining stations monthly means were calculated from 3-hourly point measurements.
[2]of difference between monthly time series and monthly-mean gridded data and daily-mean GFDL ESM2M model data.
* Stars indicate significant differences

Supplementary Table 1: LMF estimates for the time series data, the gridded observation-based data product, and the GFDL ESM2M model ensemble at different locations. For stations KNOT-K2 to CARIACO, results are obtained from roughly monthly point measurements. For the autonomous buoy stations KEO to WHOTS, the 3-hourly data was first aggregated to monthly means (column 'Time series (monthly)') or to daily-means (column 'Time series (daily mean)'; see Methods). Results for the gridded observation-based product and the GFDL ESM2M model ensemble are obtained from monthly-mean data (observation-based product) and daily-mean data (GFDL ESM2M model ensemble) for the period 1982-2019 from averaged time series over the 9 closest grid cells to the time series site. For KNOT-K2, the average of the KNOT and K2 coordinates was used for comparison to the gridded observation-based data and GFDL ESM2M model. Uncertainties are given as 90% confidence intervals and p-values are calculated for differences between the time series estimates and those for the gridded observation-based data and GFDL ESM2M model data (see Methods). All data was linearly detrended prior to the analysis**.**

	Reference	Lat (° N)	Lon (°E)	Period	Number of Data Points	Measured Variables
**KNOT**	Wakita et al.[1]	44.0	155.0	1997-2008	38	SST, S, A_T, C_T, phosphate, silicate
**K2**	Wakita et al.[2]	47.0	160.0	2001-2008	37	SST, S, A_T, C_T, phosphate, silicate
**HOT**	adapted from Dore et al.[3]	22.5	-158.0	1988-2018	299	SST, pH, S, A_T, C_T, phosphate, silicate
**BATS**	Bates and Johnson[4]	31.4	-64.1	1983-2019	456	SST, S, A_T, C_T
**ESTOC**	González Dávila: pers. comm.	29.2	-15.5	1995-2020	221	SST, S, A_T, C_T,
**137 °E 30 °N**	Sasano: pers. comm.	30.0	137.0	2000-2020	94	SST, S, A_T, C_T, phosphate, silicate
**137 °E 5 °N**	Sasano: pers. comm.	5.0	137.0	2000-2020	63	SST, S, A_T, C_T, phosphate, silicate
**Munida**	NZOA-ON data sourced from NIWA	-45.7	171.5	1998-2020	126	SST, S, A_T, pCO_2
**CARIACO**	Astor et al.[5]	10.3	-64.4	1995-2017	188	SST, S, A_T, C_T
**KEO**	Sutton et al.[6]	32.3	144.6	2007-2015	18287	SST, S, pCO_2
**Papa**	Sutton et al.[6]	50.1	-144.8	2007-2015	18039	SST, S, pCO_2
**STRATUS**	Sutton et al.[6]	-19.7	-85.6	2006-2015	19200	SST, S, pCO_2
**TAO125W**	Sutton et al.[6]	0.0	-125.0	2004-2017	15546	SST, S, pCO_2
**TAO140W**	Sutton et al.[6]	0.0	-140.0	2004-2015	14279	SST, S, pCO_2
**TAO170W**	Sutton et al.[6]	0.0	-170.0	2005-2012	11686	SST, S, pCO_2
**WHOTS**	Sutton et al.[6]	22.5	-158.0	2004-2015	23216	SST, S, pCO_2

Supplementary Table 2: Ocean stations that provide time series data used in this study. The KNOT and K2 station data were combined due to their spatial proximity. SST was measured at every station. $[H^+]$ was either calculated from measured pH at in-situ temperature (only available for HOT for 204 of 299 measurements), or calculated from measured SST, salinity (S), A_T, phosphate, silicate, and either C_T or pCO_2. For the autonomous buoy data (stations KEO to WHOTS), the raw number of 3-hourly measurements is given. Here, either monthly-mean or daily-mean values are calculated from these.

Model	Model paper	Data DOI
CanESM5	Swart et al. [7]	Swart et al.[8]; Swart et al.[9]
CNRM ESM2-1	Séférian et al.[10]	Séferian et al.[11]; Voldoire et al.[12]
GFDL CM4	Held et al.[13]	Guo et al.[14]; Guo et al.[15]
GFDL ESM4	Dunne et al.[16]	Krasting et al.[17]; John et al.[18]
IPSL CM6A-LR	Boucher et al.[19]	Boucher et al.[20]; Boucher et al.[21]
MIROC ES2L	Hajima et al.[22]	Hajima et al.[23]; Tachiiri et al.[24]
MPI ESM1-2-HR	Müller et al.[25]	Jungclaus et al.[26]; Schupfner et al.[27]
UKESM1-0-LL	Sellar et al.[28]	Tang et al.[29]; Good et al.[30]

Supplementary Table 3: CMIP6 models that were used in this study. For all models, a historical simulation over the period 1850-2014 and a simulation following the SSP5-8.5 scenario over the period 2015-2100 was used.

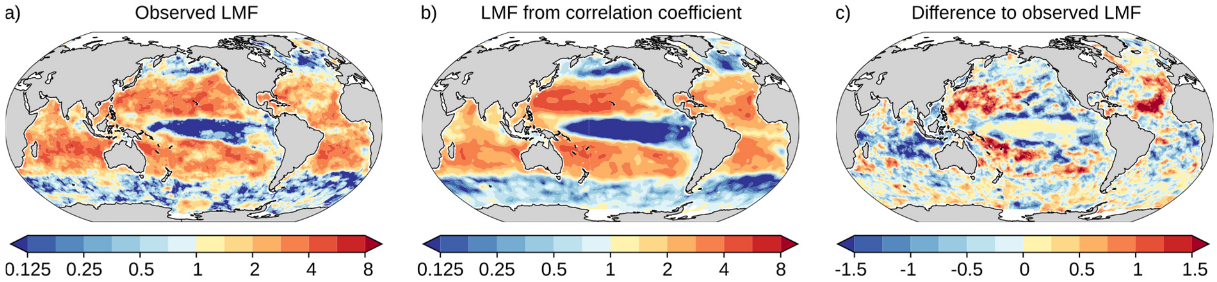

Supplementary Figure 1: Comparison of the likelihood multiplication factor with the likelihood multiplication factor estimated from correlation coefficient. (a) The likelihood multiplication factor (LMF) for MHW-OAX events for the gridded observation-based data product over the period 1982-2019 (same as in main text Fig. 1). (b) Estimated LMF from the correlation coefficient of SST and $[H^+]$ anomalies for the same data. (c) The difference between (b) and (a).

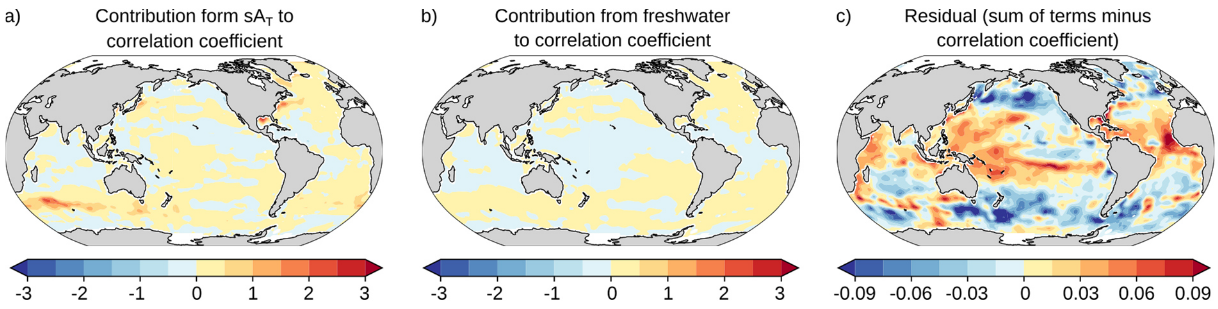

Supplementary Figure 2: Remaining terms of the correlation coefficient decomposition of SST and [H^+] anomalies. (a) Contribution from salinity-normalized alkalinity (sA_T) anomalies and (b) freshwater anomalies to the correlation coefficient of SST and [H^+] anomalies in the gridded observation-based data product over the period 1982-2019 (in correlation coefficient units; see Methods). The data was linearly detrended before analysis. (c) The residual of the decomposition, i.e. the sum of the SST, sC_T, sA_T, and freshwater contributions minus the observed correlation coefficient.

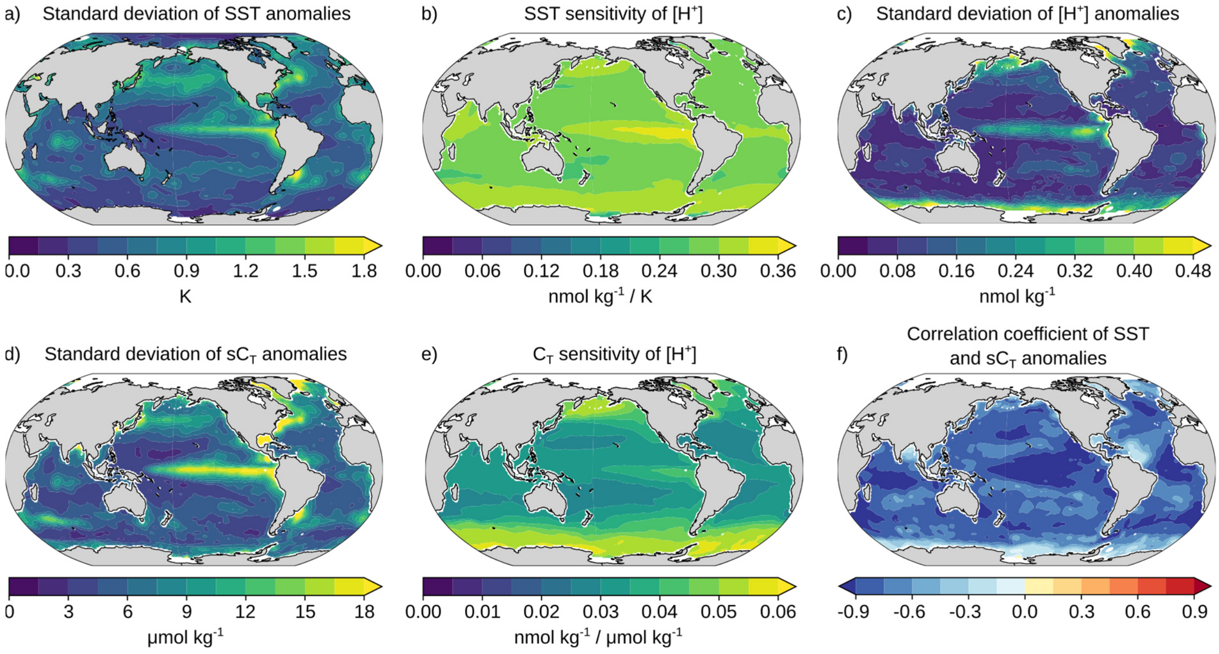

Supplementary Figure 3: Individual terms underlying the SST and sC_T contributions to the correlation coefficient of SST and [H^+] anomalies. (a) The standard deviation of SST anomalies, (b) the temperature sensitivity of [H^+], (c) the standard deviation of [H^+] anomalies, (d) the standard deviation of sC_T anomalies, € the C_T sensitivity of [H^+], and (f) the correlation coefficient of SST and sC_T anomalies. The data was linearly detrended before analysis. (a-c) form the SST contribution to the correlation coefficient of SST and [H^+] anomalies, and (c-f) form the sC_T contribution (main text Eq. 4).

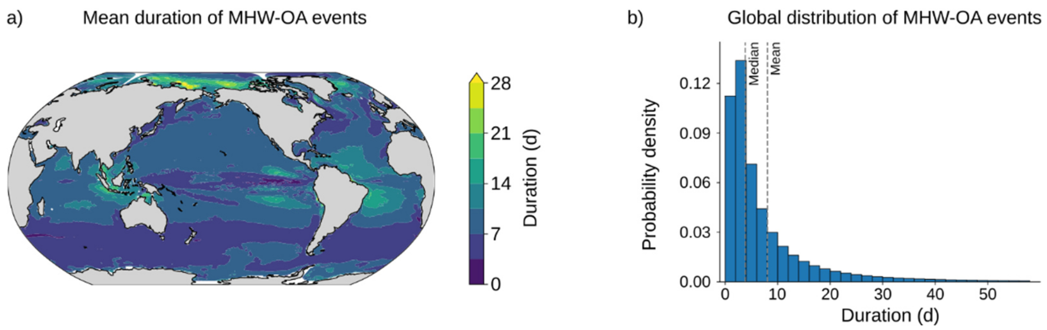

Supplementary Figure 4: Ensemble-mean duration of MHW-OAX events simulated by the GFDL ESM2M model over the period 1982-2019. (a) The mean duration of MHW-OAX events, and (b) the globally averaged and ensemble-mean histogram of event durations. The x-axis in (b) was cut for events that last more than 58 days due to the non-visible probability density. The longest MHW-OAX event lasted 433 d.

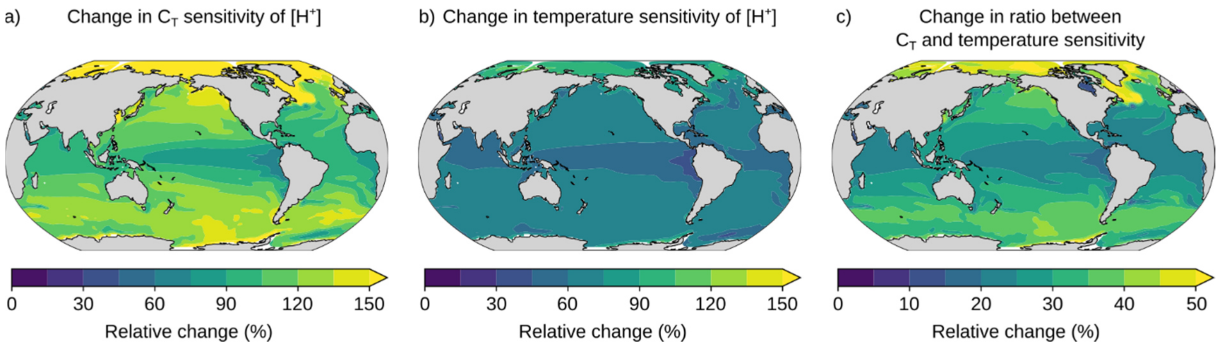

Supplementary Figure 5: Change in C_T sensitivity and temperature sensitivity of $[H^+]$ between the preindustrial conditions and 2 °C global warming level in the GFDL ESM2M model. (a) The relative change in C_T sensitivity (global average increase by 114%), (b) the relative change in temperature sensitivity (global average increase by 65%), and (c) the relative change in the ratio of C_T and temperature sensitivities (global average increase by 29%). For the mean sensitivities at 2 °C global warming level, sensitivities were calculated from the RCP8.5 ensemble mean for each day in the period 2045-2064 and then averaged. The sensitivities were calculated with *mocsy 2.0*[31] by numerical differentiation.

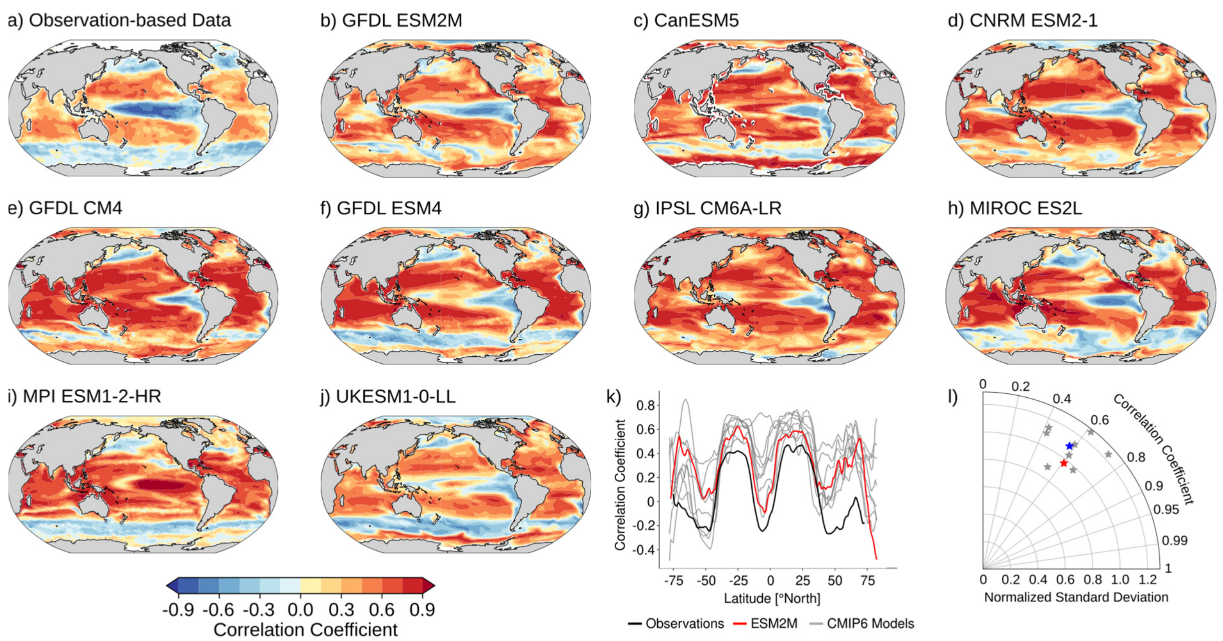

Supplementary Figure 6: Correlation coefficient of sea surface temperature and [H^+] anomalies within observation-based data product, GFDL ESM2M model and eight CMIP6 models. (a-j) Spatial patterns of correlation coefficients from gridded observation-based data, GFDL ESM2M and eight CMIP6 models (Ext. Data Table 3). (k) The zonal mean correlations from the observation-based product (black), the GFDL ESM2M model (red), and the CMIP6 models (grey). The Taylor diagram (l) displays the correlation with the observation-based pattern and the spatial standard deviation relative to that of the observation-based pattern for the GFDL ESM2M (red), CMIP6 models (grey), and CMIP6 multi-model mean (blue). The correlation coefficient was calculated from monthly-mean data over the 1982-2019 period. The data was linearly detrended prior to analysis.

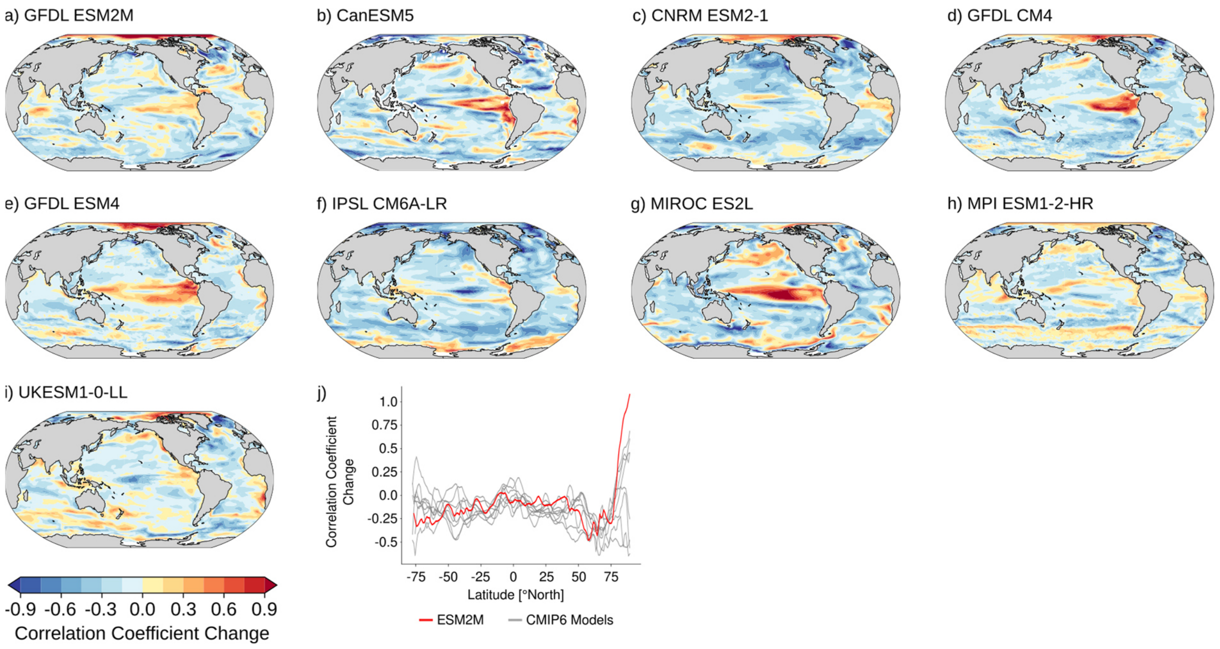

Supplementary Figure 7: Projected change in correlation coefficient of temperature and [H^+] anomalies between 1982-2019 and 2071-2100. Spatial patterns of correlation coefficient changes for (a) the GFDL ESM2M model under RCP8.5 and (b-i) eight CMIP6 models under SSP5-8.5 (Ext. Data Table 3). (j) The zonal mean correlation coefficient change in the GFDL ESM2M model (red) and the CMIP6 models (grey). In the GFDL ESM2M model, the correlation coefficient decreases globally by -0.11 between the preindustrial period and 2071-2100 under RCP8.5. The CMIP6 models simulate a global decrease of -0.08 to -0.20 between the preindustrial period and 2071-2100 under SSP5-8.5.

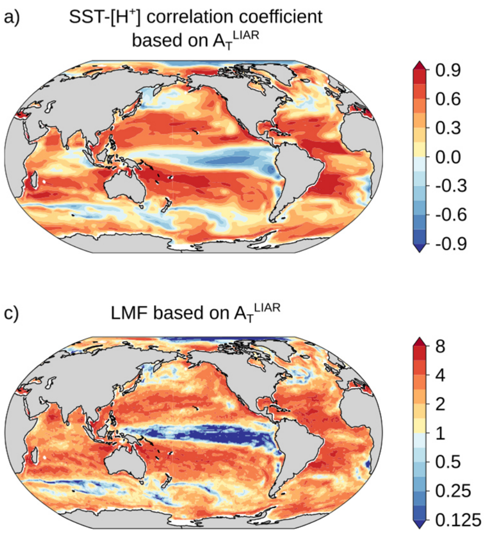

Supplementary Figure 8: The correlation coefficient of SST and [H^+] within the GFDL ESM2M model when A_T is estimated from temperature and salinity using the *LIARv2* algorithm. (a) The correlation coefficient of SST and [H^+] anomalies obtained by calculating A_T from simulated SST and salinity using *LIARv2* (A_T^{LIAR}) and then calculating [H^+] from simulated SST, salinity, $p$$CO_2$, and LIARv2 A_T using CO2SYS. (b) The difference between the correlation coefficient based on A_T^{LIAR} (panel a) and the simulated correlation coefficient. (c) The LMF with [H^+] calculated from A_T^{LIAR} as in panel a), and (d) the difference between (c) and the simulated LMF. Data is shown for one ensemble member over the period 1982-2019 using monthly mean data.

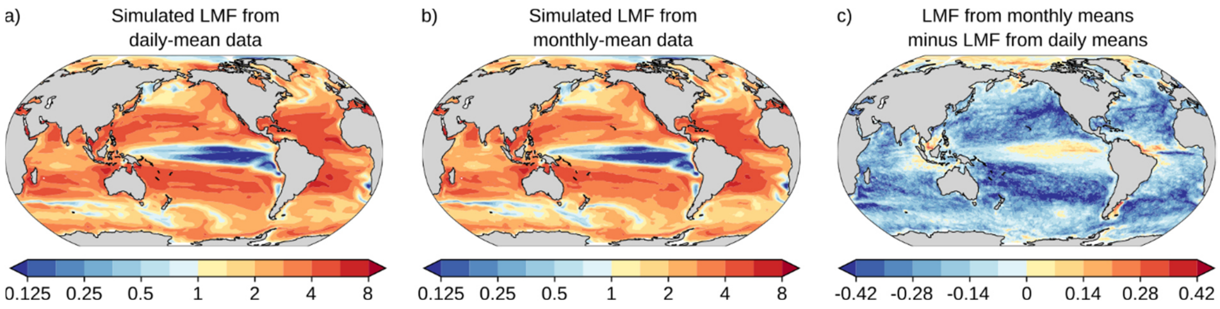

Supplementary Figure 9: Simulated ensemble-mean LMF over the period 1982-2019 from daily-mean and monthly-mean data as simulated by the GFDL ESM2M model. Simulated LMF from daily-mean data (a), from monthly-mean data (b), and the difference between the LMF from monthly-mean and daily-mean data (c).

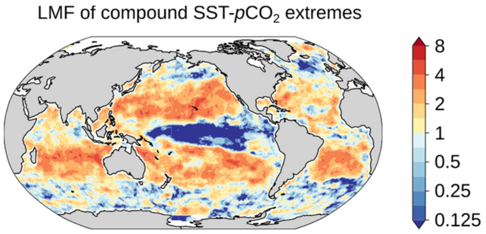

Supplementary Figure 10: The observation-based likelihood multiplication factor of compound MHWs and extremes in *p*CO_2 over the years 1982 to 2019. Map of the likelihood multiplication factor (LMF) based on the global monthly observation-based SST and surface *p*CO_2 data (*MPI-SOMFNN* product[32]). Warm colors indicate LMF > 1 and cold colors indicate LMF < 1. The data was linearly detrended prior to analysis.

REFERENCES

1. Wakita, M., Watanabe, S., Murata, A., Honda, M. C. & Tsurushima, N. Hydrographic Data Report at Station KNOT. http://www.godac.jamstec.go.jp/k2/ (2011).
2. Wakita, M., Watanabe, S., Murata, A. & Honda, M. C. Hydrographic Data Report at Station K2. http://www.godac.jamstec.go.jp/k2/ (2011).
3. Dore, J. E., Lukas, R., Sadler, D. W., Church, M. J. & Karl, D. M. Physical and biogeochemical modulation of ocean acidification in the central North Pacific. *Proc. Natl. Acad. Sci.* **106**, 12235–12240 (2009).
4. Bates, N. R. & Johnson, R. J. Acceleration of ocean warming, salinification, deoxygenation and acidification in the surface subtropical North Atlantic Ocean. *Commun. Earth Environ.* **1**, 33 (2020).
5. Astor, Y. M. *et al.* Interannual variability in sea surface temperature and fCO2 changes in the Cariaco Basin. *Deep Sea Res. Part II Top. Stud. Oceanogr.* **93**, 33–43 (2013).
6. Sutton, A. J. *et al.* Autonomous seawater pCO2 and pH time series from 40 surface buoys and the emergence of anthropogenic trends. *Earth Syst. Sci. Data* **11**, 421–439 (2019).
7. Swart, N. C. *et al.* The Canadian Earth System Model version 5 (CanESM5.0.3). *Geosci. Model Dev.* **12**, 4823–4873 (2019).
8. Swart, N. C. *et al.* CCCma CanESM5 model output prepared for CMIP6 CMIP historical. (2019) doi:10.22033/ESGF/CMIP6.3610.
9. Swart, N. C. *et al.* CCCma CanESM5 model output prepared for CMIP6 ScenarioMIP ssp585. (2019) doi:10.22033/ESGF/CMIP6.3696.
10. Séférian, R. *et al.* Evaluation of CNRM Earth System Model, CNRM-ESM2-1: Role of Earth System Processes in Present-Day and Future Climate. *J. Adv. Model. Earth Syst.* **11**, 4182–4227 (2019).
11. Séférian, R. CNRM-CERFACS CNRM-ESM2-1 model output prepared for CMIP6 CMIP historical. (2018) doi:10.22033/ESGF/CMIP6.4068.
12. Voldoire, A. CNRM-CERFACS CNRM-ESM2-1 model output prepared for CMIP6

ScenarioMIP ssp585. (2019) doi:10.22033/ESGF/CMIP6.4226.

13. Held, I. M. *et al.* Structure and Performance of GFDL's CM4.0 Climate Model. *J. Adv. Model. Earth Syst.* **11**, 3691–3727 (2019).

14. Guo, H. *et al.* NOAA-GFDL GFDL-CM4 model output historical. (2018) doi:10.22033/ESGF/CMIP6.8594.

15. Guo, H. *et al.* NOAA-GFDL GFDL-CM4 model output prepared for CMIP6 ScenarioMIP ssp585. (2018) doi:10.22033/ESGF/CMIP6.9268.

16. Dunne, J. P. *et al.* The GFDL Earth System Model Version 4.1 (GFDL-ESM 4.1): Overall Coupled Model Description and Simulation Characteristics. *J. Adv. Model. Earth Syst.* **12**, e2019MS002015 (2020).

17. Krasting, J. P. *et al.* NOAA-GFDL GFDL-ESM4 model output prepared for CMIP6 CMIP historical. (2018) doi:10.22033/ESGF/CMIP6.8597.

18. John, J. G. *et al.* NOAA-GFDL GFDL-ESM4 model output prepared for CMIP6 ScenarioMIP ssp585. (2018) doi:10.22033/ESGF/CMIP6.8706.

19. Boucher, O. *et al.* Presentation and Evaluation of the IPSL-CM6A-LR Climate Model. *J. Adv. Model. Earth Syst.* **12**, e2019MS002010 (2020).

20. Boucher, O. *et al.* IPSL IPSL-CM6A-LR model output prepared for CMIP6 CMIP historical. (2018) doi:10.22033/ESGF/CMIP6.5195.

21. Boucher, O. *et al.* IPSL IPSL-CM6A-LR model output prepared for CMIP6 ScenarioMIP ssp585. (2019) doi:10.22033/ESGF/CMIP6.5271.

22. Hajima, T. *et al.* Development of the MIROC-ES2L Earth system model and the evaluation of biogeochemical processes and feedbacks. *Geosci. Model Dev.* **13**, 2197–2244 (2020).

23. Hajima, T. *et al.* MIROC MIROC-ES2L model output prepared for CMIP6 CMIP historical. (2019) doi:10.22033/ESGF/CMIP6.5602.

24. Tachiiri, K. *et al.* MIROC MIROC-ES2L model output prepared for CMIP6 ScenarioMIP ssp585. (2019) doi:10.22033/ESGF/CMIP6.5770.

25. Müller, W. A. *et al.* A Higher-resolution Version of the Max Planck Institute Earth System

Model (MPI-ESM1.2-HR). *J. Adv. Model. Earth Syst.* **10**, 1383–1413 (2018).

26. Jungclaus, J. *et al.* MPI-M MPI-ESM1.2-HR model output prepared for CMIP6 CMIP historical. (2019) doi:10.22033/ESGF/CMIP6.6594.

27. Schupfner, M. *et al.* DKRZ MPI-ESM1.2-HR model output prepared for CMIP6 ScenarioMIP ssp585. (2019) doi:10.22033/ESGF/CMIP6.4403.

28. Sellar, A. A. *et al.* UKESM1: Description and Evaluation of the U.K. Earth System Model. *J. Adv. Model. Earth Syst.* **11**, 4513–4558 (2019).

29. Tang, Y. *et al.* MOHC UKESM1.0-LL model output prepared for CMIP6 CMIP historical. (2019) doi:10.22033/ESGF/CMIP6.6113.

30. Good, P. *et al.* MOHC UKESM1.0-LL model output prepared for CMIP6 ScenarioMIP ssp585. (2019) doi:10.22033/ESGF/CMIP6.6405.

31. Orr, J. C. & Epitalon, J.-M. Improved routines to model the ocean carbonate system: mocsy 2.0. *Geosci. Model Dev.* **8**, 485–499 (2015).

32. Landschützer, P., Gruber, N. & Bakker, D. C. E. Decadal variations and trends of the global ocean carbon sink. *Glob. Biogeochem. Cycles* **30**, 1396–1417 (2016).
